# Spina Bifida

**Published:** 1890-07-26

**Authors:** 


### SURGERY.

### SPINA BIFIDA.

If we recollect right a committee of the Clinical Society, in 1885, reported that the term spina bifida was properly applied to define certain congenital malformations of the vertebral column, with protrusions of some of its contents in the form of a fluid tumour. With very rare exceptions the malformation affects the neural arches of the vertebræ, and the tumour projects posteriorly. Occasionally the bodies of the vertebræ are wanting, and the tumour then protrudes anteriorly into the thorax, abdomen or pelvis. Authors describe three varieties: (1) Protrusion of membranes only; (2) protrusion of the membranes, together with the spinal cord and its appertaining nerves; (3) protrusion of the membranes, together with the spinal cord, the central cavity of which is dilated so as to form the sac cavity, the innermost lining being constituted by the expanded and atrophied substance of the cord. To these varieties the terms "simple meningocele," "meningo-myelocele," and "syringo-myelocele," have been given. Although spina bifida is not a very common condition, it still presents sufficiently often to demand attentive consideration. A tumour of the kind is usually of a round or oval shape, varying in size from that of a hazel nut to that of an orange. Sometimes, however, it is large enough to delay delivery. Last year, Mr. G. H. A. Da Costa reported a case where perforation of the tumour was required to accomplish delivery. It most commonly occurs in the lumbar or sacral region. In some cases the tumour has appeared bilobed. It usually diminishes on pressure, and sometimes it may be made to completely disappear. The bony margin of the cleft is often distinctly felt. In some cases fluctuation may be rendered apparent by alternately compressing the sac and the skull, when spasms of the limbs may be caused. It is frequently complicated by club foot, hydrocephalus, or paralysis of the lower extremities. In November last, Mr. J. J. Clarke showed the members of the Harveian Society a case of myelocele with equino-varus of both feet. Much discussion has taken place as to the proper method of dealing with these cases. Some have advised injections, especially of Morton's fluid, composed of iodine gr. ix., iodide of potassium ʒ½ and glycerine ℥i. The committee of the Clinical Society collected 71 cases treated by this means, of whom 35 recovered, 27 died, 4 were relieved, and 5 experienced no benefit. Simple tapping repeatedly performed, the orifice being closed after fluid has drained away, has sometimes succeeded. Tapping and draining have not proved so successful, for the means used for draining have been considered to set up septic irritation. The sac has also been excised, after which recoveries have been reported. Ligatures have also been applied and kept on till the tumours sloughed off. Constant pressure by some mechanical contrivance has been used.

We think, however, that what Holmes says in his "Surgical Treatment of Children's Diseases" is correct. It is there observed that, in some forms of the disease, spina bifida is manifestly incurable, the mechanical conditions of the disease not allowing any interference with the part, except at the cost of speedy death. No treatment will be likely to have any effect on the tumour, which is not sufficiently active to set up inflammation of the inner surface of the sac enough to obliterate its neck, and this inflammation extending, may be fatal. If the cord passes through the tumour, the latter cannot be severed without dividing the cord, nor closed by inflammation without inducing fatal softening. On the other hand, the rarer forms of spina bifida, in which there are none of the nervous structures in the sac, do not usually cause symptoms calling for interference. Under these circumstances few cases present, in which the prudent surgeon will do more than recommend the wearing of some defence to the tumours; for it is extremely difficult to determine to what extent the nervous structure of the cord is implicated. If affected to any extent, there will be convulsions, paraplegia, paralysis of sphincters, to a greater or lesser degree. It has been sought to connect the condition of the spinal cord or nerves, with the position and size of the tumour. But the practical point is, that in cases where the opening into the spinal cord is free and direct, there is very great probability of the cord or nerves being in the sac. It also may be assumed, that if the tumour has a long and narrow pedicle, the cord itself does not pass through it. But there is no possibility of absolutely distinguishing a case in which the cord or nerves may be implicated, from one in which they are free. It has been stated that evidence of the cord in the sac is afforded by umbilication, or a longitudinal furrow on the surface, and the appearance of cord-like bands when the sac is thin enough to transmit light. But these tests are not sufficient. Sometimes the cord forms part of the sac walls, its dilated central canal containing the fluid (syringo-myelocele). Again, the cauda-equina may pass through the centre of the sac, or be spread out on its walls. Under such circumstances and with the fact before us that the duration of life of those affected, may extend to 20 or 30 years, or even in one case reported to 73, it is no matter of surprise that many surgeons have advocated non-interference. A person with spina bifida at 32 years of age was once cut for stone, and lived long afterwards. Spontaneous rupture has also terminated satisfactorily. Mr. Jacobson has thus summed up the causes of failure after attempting the radical cure of spina bifida: 1, leakage and septic meningitis; 2, convulsions and rapid death; 3, hydrocephalus; 4, no result, the swelling progressing unaltered.

In a recent number of the *Philadelphia Therapeutic Gazette*, Dr. E. P. Hurd gives a paper on the operative treatment of spina bifida, and describes an operation he performed. A child seven months old was brought with a congenital swelling in the lumbo-sacral region. The tumour was entirely covered by thin skin, being soft, elastic, and fluctuating. The shape was round, but terminating in a pyriform point on the left side. The tumour seemed to be connected with the bony substance beneath by a large base, and could not be moved to either side. Neither pressure, nor cries, nor movement of the child made any difference in the volume of the swelling. A diagnosis of meningo-myelocele was made, with a small foramen of communication with the spinal canal. With a hypodermic needle some liquid was drawn off, clear and thin, and not coagulating by heat; neither was there sugar. Dr. Hurd was doubtful about the expediency of any operation except Morton's, to which the parents would not accede. Some months after the patient was brought again, with the tumour increased to the size of a quart bowl. Other medical men who saw the case for the first time were inclined to regard the swelling as a simple cyst, unconnected with the spinal cord or canal, and advised immediate removal. Aspiration first was decided upon, which was performed at the most dependent position, and all the liquid contents withdrawn. Symptoms of collapse followed, but the child rallied in the course of an hour. Afterwards an examination led to the belief that there was nothing to indicate the swelling was more than a simple cryst, and it was decided to dissect it out. Ether was administered, and the inside of the sac freely exposed, when the nerves forming the cauda-equina were seen spreading over the surface of the white shining membrane constituting the sac. A small loss of substance in the arch of the last lumbar vertebræ was also discovered. Here a small foramen was visible, and a probe easily passed into the spinal canal. But a menace of convulsions was observed. Therefore "we hastily removed a considerable portion of the surplus integument of the sac, and brought the edges of the incision together with a continuous catgut suture, painting flexible collodion over the whole." Two days afterwards it was reported that the sac had filled and burst, and it was found about a pint of liquid had drained away. It was now certain that the sac would continue secreting, which would soon enfeeble the child. With consent of the parents, ether was again administered, the previous line of incision was opened up, and the cavity sponged with sublimate solution. "The glistening sac, with its nerve filaments and median core, was dissected or torn from its subjacent tissues, till we had the entire membrane in our hands down to the foramen of entrance into the vetebral canal. A cat-gut ligature was passed round the neck of the sac, firmly tied, and then the whole sac was excised. Iodoform was dusted over the raw surfaces, the edges of the wound stitched together, and a compress and bandage applied. The subsequent progress of the case was most satisfactory. Dr. Hurd truly remarks that the operation is sufficiently rare in surgery to justify his giving the history of it. And Dr. Hurd also observes that Duplay and Virchow have stated there almost always exists a depression on the surface of the tumour or a patch of thickened skin, which was present in the case at first, and should have been sufficient to prevent the second diagnosis of simple cyst. But he did not duly weigh the importance of this sign. In some remarks on the case, Dr. Hurd observes that excision of the sac was practised by Dubourgh more than fifty years ago; and Chelius says the operation is required when the swelling and opening are of small size, and the child's health otherwise good. Dr. Hurd further observes that the result of this case demonstrates two points—first, that the loss of the fluid contents of the sac in a lumbo-sacral spina bifida is not necessarily a serious matter; secondly, that the cauda-equina is of no special consequence in the economy, and may be removed with impunity when part of a spinal hernia.

Notwithstanding that so many instances of recovery after various operations have been recorded, we certainly incline to the palliative treatment. This consists of moderate compression over the tumour. If, however, the condition produces symptoms of paralysis of sphincters, or other grave complications, we think operative procedure is justified. A consideration of the various measures proposed leads to the belief that injections of Morton's fluid will be most likely to prove satisfactory. Some time ago Mr. Gould gave his views as to the value of iodo-glycerine, stating that it does not mix with the cerebro-spinal fluid, but sinks, its action, therefore being more potent on the lower part of the sac. It was, we believe, Dr. Branard, of Chicago, who first suggested, in1848, the injection of a mixture of iodine and iodide of potassium in water. The improvement of substituting glycerine was Dr. James Morton's. The treatment certainly commends itself for simplicity and ease of application. A small quantity of fluid is drawn off by subcutaneous puncture. About half a drachm of the iodo-glycerine is injected, the puncture is sealed with collodion, pressure is applied, and when the sac refills the injection is repeated.

